# A prognostic model for development of significant liver fibrosis in HIV-hepatitis C co-infection

**DOI:** 10.1371/journal.pone.0176282

**Published:** 2017-05-03

**Authors:** Nasheed Moqueet, Cynthia Kanagaratham, M. John Gill, Mark Hull, Sharon Walmsley, Danuta Radzioch, Sahar Saeed, Robert W. Platt, Marina B. Klein, for the Canadian Co-infection Cohort Study (CTN 222)

**Affiliations:** 1 Department of Epidemiology, Biostatistics, and Occupational Health, McGill University, Montreal, QC, Canada; 2 Department of Medicine and Department of Human Genetics, McGill University, Montreal, QC, Canada; 3 Southern Alberta HIV Clinic, Calgary, Alberta, Canada; 4 BC Centre for Excellence in HIV/AIDS, St. Paul's Hospital, Vancouver, BC, Canada; 5 Toronto General Research Institute, University Health Network, University of Toronto, Toronto, ON, Canada; 6 Division of Infectious Diseases and Chronic Viral Illness Service, McGill University Health Centre, Montreal, QC, Canada; University of Cincinnati College of Medicine, UNITED STATES

## Abstract

**Background:**

Liver fibrosis progresses rapidly in HIV-Hepatitis C virus (HCV) co-infected individuals partially due to heightened inflammation. Immune markers targeting stages of fibrogenesis could aid in prognosis of fibrosis.

**Methods:**

A case-cohort study was nested in the prospective Canadian Co-infection Cohort (n = 1119). HCV RNA positive individuals without fibrosis, end-stage liver disease or chronic Hepatitis B at baseline (n = 679) were eligible. A random subcohort (n = 236) was selected from those eligible. Pro-fibrogenic markers and Interferon Lambda (IFNL) rs8099917 genotype were measured from first available sample in all fibrosis cases (APRI ≥ 1.5 during follow-up) and the subcohort. We used Cox proportional hazards and compared Model 1 (selected clinical predictors only) to Model 2 (Model 1 plus selected markers) for predicting 3-year risk of liver fibrosis using weighted Harrell’s C and Net Reclassification Improvement indices.

**Results:**

113 individuals developed significant liver fibrosis over 1300 person-years (8.63 per 100 person-years 95% CI: 7.08, 10.60). Model 1 (age, sex, current alcohol use, HIV RNA, baseline APRI, HCV genotype) was nested in model 2, which also included IFNL genotype and IL-8, sICAM-1, RANTES, hsCRP, and sCD14. The C indexes (95% CI) for model 1 vs. model 2 were 0.720 (0.649, 0.791) and 0.756 (0.688, 0.825), respectively. Model 2 classified risk more appropriately (overall net reclassification improvement, p<0.05).

**Conclusions:**

Including IFNL genotype and inflammatory markers IL-8, sICAM-1, RANTES, hs-CRP, and sCD14 enabled better prediction of the 3-year risk of significant liver fibrosis over clinical predictors alone. Whether this modest improvement in prediction justifies their additional cost requires further cost-benefit analyses.

## Introduction

Liver disease has become one of leading non-AIDS causes of death among HIV-infected individuals in the developed world, mainly due to co-infection with hepatitis C (HCV). While HIV treatment improves outcomes in HCV co-infection [1, 2], compared to HCV mono-infected individuals, liver fibrosis progression remains accelerated, leading to cirrhosis, hepatocellular carcinoma or end-stage liver disease (ESLD) [3, 4]. Reasons for this acceleration include biological factors and possibly HIV therapy-related toxicity. HIV itself suppresses the immune response to HCV [3, 5], triggering a cycle where inflammatory and fibrogenic cells continually stimulate each other, distorting the hepatic architecture, eventually leading to fibrosis. Liver fibrosis progression is thus caused by heightened inflammation rather than direct HCV replication.

In Canada, the majority of the HIV-HCV co-infected population is made up of current or former injection drug users for whom treatment access and adherence may be challenging. While international clinical guidelines recognize that co-infected individuals should be prioritized for HCV treatment [6, 7], the high cost of treatment (between $50,000 and $120,000 for a course of the new direct-acting antivirals or DAAs) in Canada [8, 9] has meant that reimbursement by public and private payers has been restricted to those with advanced fibrosis (METAVIR stage F2 and higher), based on liver biopsy and increasingly by transient elastography or use of noninvasive indices such as aspartate aminotransferase (AST) to platelet ratio index (APRI) and Fibrosis-4 (FIB-4) [10]. Given this restriction, earlier indicators of fibrosis could be helpful in identifying persons at higher risk for liver disease progression in order to target effective intervention and treatment strategies in a cost-effective manner.

The principal risk factors associated with fibrosis progression include alcohol intake (>50 g/day), infection with HCV genotype 3, male sex, excess weight, liver steatosis, presence of metabolic syndrome and/or type II diabetes, host genetic factors such as single nucleotide polymorphisms (SNPs) in the Interferon Lambda (IFNL) gene, hepatitis B co-infection, immunodeficiency related to HIV or the use of immunosuppressant drugs, and HIV therapy interruption [11–15].

We hypothesized that the addition of genetic markers associated with fibrosis progression and a panel of immune markers (summarized in S1 Table) representative of the underlying inflammatory mechanisms would improve prediction of fibrosis risk beyond traditional clinical risk factors alone and would thus be of value in optimal use of new and expensive HCV therapies.

## Methods

### Source population

The Canadian Co-infection Cohort is an open prospective cohort of HIV-HCV co-infected patients recruited from 18 centers across Canada[16]. It has been approved by the following: the community advisory committee of the CIHR-Canadian HIV Trials Network, the Biomedical B Research Ethics Board of the McGill University Health Centre (BMB-06-006t and BMC-07-004), the UBC-Providence Health Care Research Ethics Board (H08-00474), the Institutional Review Board Services, Regina Qu’Appelle Health Region Research Ethics Board (REB-14-70), the Conjoint Health Research Ethics Board of the University of Calgary (20931), the Nova Scotia Health Research Ethics Board (CDHA-RS2007-118), the Windsor Regional Hospital Research Ethics Board (07-122-17), the Veritas Independent Review Board, the Hamilton Integrated Research Ethics Board (06–397), the Comité d'éthique de la recherche du CHUM (2003–1582, SL 03.008-BSP), the Comité d'éthique de la recherche du CHU de Québec-Université Laval (C11-12-153), the Sunnybrook Health Sciences Centre Research Ethics Board (252–2008), the Research Ethics Board of Health Sciences North (605), the University Health Network Research Ethics Board (06-0629-BE), the Ottawa Health Science Network Research Ethics Board (2007229-01H) and the Biomedical Research Ethics Board (12–178). Patient records/information was anonymized and de-identified prior to analysis. All patients gave written informed consent before undergoing an initial evaluation and were followed at study visits every six months.

The CCC represents approximately 23% of the co-infected population under care. At every study visit, socio-demographic and behavioural information was collected using validated questionnaires, along with plasma, serum and peripheral blood mononuclear cells (PBMC). For our study, we included data from 1119 patients collected up until July 2012.

To be included in the CCC, patients must be over 16 years or older, give informed consent, be HIV infected (confirmed via ELISA with Western blot), and have HCV infection or evidence of HCV exposure (HCV-antibody positive by ELISA with recombinant immunoblot assay II (RIBA II) or enzyme immunoassay (EIA) or if serologically false negative, HCV–RNA-positive).

### Study population

For our study, HCV RNA-negative participants or those who had significant fibrosis (APRI≥1.5), end-stage liver disease or chronic Hepatitis B at study entry were excluded, as were individuals on HCV treatment. HCV RNA was measured using qualitative tests (COBAS AMPLICOR HCV Test, version 2.0, Roche Diagnostics, Hoffmann-La Roche Ltd, Laval, Canada, lower limit of detection <50 IU ml-^1^) and was available at most visits. Presence of Hepatitis B surface antigen was used to determine Hepatitis B chronicity.

**Fig 1:**
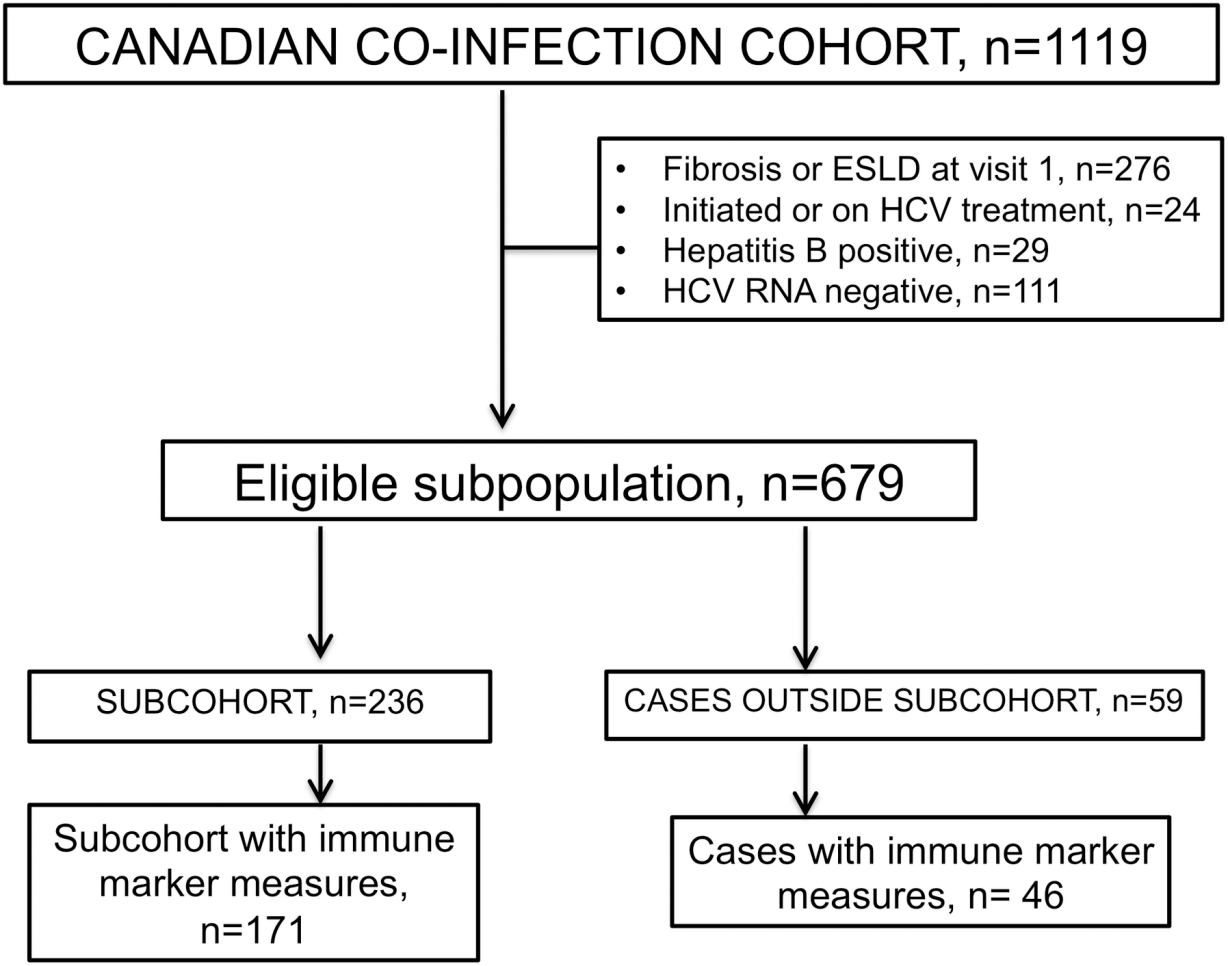
Source and study population for developing a prognostic model for significant liver fibrosis. Abbreviations: ESLD, end-stage liver disease; HCV, Hepatitis C virus.

As immune and genetic markers were not measured during regular CCC visits, we used a case cohort study design as an economical way to gather this information. From an eligible study sample of n = 679 (Fig 1), a random subsample or “subcohort” was selected from the population at entry, to provide comparison observations for each event of significant liver fibrosis, occurring during study follow-up. Because the subcohort was a representation of the full cohort, it also contained a few incident cases.

### Outcome measure

Progression to significant liver fibrosis (METAVIR stage F2-F4) was defined by an APRI≥1.5. APRI is calculated as follows: [(AST/upper limit of normal)/platelet count (10^9^ /L)] x 100. It consists of routinely available and non-invasive measures that were available for almost every visit. An APRI cutoff of 1.5 or higher has been validated against liver biopsies in our study population for detection of significant liver fibrosis (METAVIR stages F2-F4) with a sensitivity of 52%, specificity and positive predictive value (PPV) of over 99% and an AUC of 0.85 ± 0.06.[17] Despite the lower sensitivity, APRI cutoffs of 1.5 and 2 have also been shown to be associated with cirrhosis, other adverse liver and clinical outcomes, and death in our study cohort [18] as well as in others [19, 20].

### Clinical predictors

All values were time-fixed at first available visit and distribution was evaluated in the subcohort and compared to the full eligible study sample to mimic how a predictive score would be applied in practice. Predictors considered included age; sex; ethnicity; alcohol use; body mass index (BMI); HIV viral load; CD4 count; and baseline APRI. Variables included in fibrotic staging indexes from other studies such as total bilirubin [21, 22] or gamma-glutamyltransferase (GGT) [21–23] were also considered.

### Markers of interest

Of interest to us was the genetic marker at IFNL SNP rs8099917, which has been linked with elevated histological inflammatory activity [14, 24–28] as well as Natural Killer (NK) cell activation, resulting in cell death of infected liver cells and a pro-inflammatory environment [29, 30]. We chose the following immune markers based on their specific roles in liver fibrosis development (S1 Table): the cytokines transforming growth factor beta 1 (TGF-β1) and tumor necrosis factor alpha (TNF-α); the chemokines interleukin-8 (IL-8); monocyte chemotactic protein-1 (MCP-1 or CCL2); macrophage inflammatory protein 1 (MIP1α or CCL3; MIP1β or CCL4); Regulated upon Activation, Normal T cell Expressed and Secreted protein (RANTES or CCL5); CXCL9; and CXCL11; endothelial activation markers soluble Intercellular Adhesion Molecule 1 (sICAM-1) and soluble Vascular Cell Adhesion Molecule 1 (sVCAM-1); high-sensitivity C-reactive protein (hsCRP); and soluble CD14 (sCD14), a marker of microbial translocation [31, 32].

Immune markers were measured in patients with available samples from visit 1 or 2 from all the cases and the subcohort (n = 171 from subcohort and 46 non-subcohort cases, Fig 1). Frozen, never thawed plasma or serum samples were used. After thawing on ice, viral activity was inactivated (with a 0.5% sample concentration of Triton X-100 and 30 minute incubation at room temperature). Samples were aliquoted, refrozen and thawed on ice before running with a commercial assay from Millipore on a MAGPIX instrument (Millipore Corporation, Billerica MA) according to the manufacturer’s instructions. Samples were diluted according to kit recommendations: 3-plex (RANTES, sICAM1, sVCAM1; TGF- β1, 2, 3), 6-plex (IL6, IL8, MCP1, MIP1α, MIP1β, and TNFα) and 2-plex (CXCL9 and CXCL11). Standards were prepared in the same background as samples.

Commercially available ELISA kits were used to measure plasma and serum levels of soluble CD14 (sCD14, R&D Systems, Minneapolis, MN, USA) with dilutions of 1:300. High-sensitivity C-reactive protein (hsCRP) was tested based on manufacturer’s instructions, using immunoassay kits from Synchron LX 20 PRO (Beckman Coulter, Ontario, Canada). Hyaluronic acid, another direct marker of fibrogenesis [33] that has been included in other fibrotic indexes [22, 34] was also measured with a 1:30 dilution using the Hyaluronan Quantikine ELISA kit (R&D Systems, Minneapolis, MN, USA).

To measure IFNL genotypes, never thawed plasma and serum samples were processed using a real-time PCR assay developed by the Bay Area Genetic Lab (BAGL, Ontario, Canada), as previously described [35].

### Statistical analysis

#### Survival analysis

The subcohort (n = 236) was generated with a random sampling fraction of 0.45 and included 54 cases. Cases that were not in the subcohort (n = 59) entered via delayed entry. The time axis was follow-up time in study. Cox proportional hazards was used for analysis, with robust variance and Barlow weights to account for the case cohort design [36].

Descriptive analysis was conducted in the subcohort using box plots, histograms, correlation matrices with Spearman’s correlation coefficient, scatter plots and Q-Q plots. Values of the markers that were near the limits of detection were assigned the lowest detectable value.

#### Predictor selection and functional form

Predictors were selected based on their availability to physicians, strength of correlation with each other [37], magnitude of associations in univariable analyses, ability to improve model fit as indicated by the Akaike Information Criterion (AIC) and ability to maximize discrimination as measured by Harrell’s C [38]. For immune markers, variables were also included in models if they captured a different stage of the underlying etiology of fibrosis development or were linked to fibrogenesis in the literature.

Univariable and multivariable analyses were conducted on untransformed variables, as well as after log-transformation or using median or quartile distributions of the immune markers. Continuous variables were centered at their mean values. Log transformation was used for all included continuous variables such as baseline APRI and immune markers IL-8, sICAM-1, RANTES, hsCRP, and sCD14 but not for age, which was modeled as a restricted cubic spline with 3 knots at the 10th, 50th and 90^th^ percentiles corresponding to ages 33, 44, and 54. HIV viral load was dichotomized (undetectable or not at ≤ 50 copies/ml), as were alcohol use (currently drinking or not); HCV genotype (3 vs. non-3, i.e. types 1, 2, and 4); and host IFNL genotype rs8099917 (TT vs. non-TT).

Proportional hazards were assessed using the using Stata command–stphtest, detail- which uses scaled Schoenfeld residuals to check if proportional hazards holds globally and for included predictors.

#### Predictive accuracy

Discrimination, calibration and changes in reclassification were compared between Model 1 (selected clinical predictors only) and Model 2 (clinical predictors from Model 1 plus selected genetic and immune markers) for predicting 3-year risk of significant liver fibrosis.

Discrimination was measured with a weighted [39] Harrell’s C or concordance index using Stata command–somersd- with robust jackknife estimator for standard errors [38]. A C-index value of 1 indicates perfect discrimination, while 0.5 means no better than random guessing. Calibration was assessed statistically (Hosmer-Lemeshow statistic and the Gronnesby and Borgan (GB) test) [40], and graphically with the Stata command–stcoxgrp using imputed data [41].

Change in reclassification was measured by the net reclassification improvement (NRI) summary index [42]. We calculated both the category-based and the continuous NRIs. For the category-based NRI, we used 3 clinically relevant risk categories: low risk, < = 10%; medium risk, >10–25%; and high risk, >25%. The categories were determined based on estimates of mortality from liver disease in those with chronic HCV infection from published reports [43] as well as opinions of knowledgeable hepatologists and clinicians. For the continuous NRI, no categories were needed and any upward or downward movement in risk was considered, regardless of magnitude.

The models were internally validated using bootstrapping. All analyses were conducted using Stata 12.

#### Multiple imputation

Missingness for plasma samples and other variables was assumed to be at random. Multiple Imputation by Chained Equations (MICE) was used on the full cohort to account for all missing data, using all the predictors in the final models, all the immune markers, as well as variables that were possibly related to the reasons for missingness.

We compared predictive accuracy after using unweighted Cox proportional hazards regression on the imputed full cohort data [44].

## Results

The subcohort selected was representative of both the CCC and the eligible subpopulation from which it was derived (Tables 1 and 2). A notable difference was in the median APRI score at baseline, which was much higher in the CCC overall, likely due to the 276 prevalent cases of significant liver fibrosis and end-stage liver disease (ESLD) that were excluded. The majority of the study participants were white males with a median age of 44 years; half had been infected with HCV for 18 years and almost half reported drinking alcohol (Table 1). Most were receiving HIV antiretroviral therapy and had well-controlled HIV with good CD4 recovery (>350 cells/μl) and undetectable HIV viral load.

**Table 1:** Baseline characteristics of the source, study and analytic populations.

Characteristic	CCC, n = 1119	Eligible cohort, n = 679	Subcohort, n = 236	Cases outside subcohort, n = 59
Age at baseline, years	45 (39–50)	44 (39–49)	44 (39–49)	44 (39–49)
White	855 (77)	521 (77)	182 (78)	48 (81)
Female	291 (26)	187 (28)	70 (30)	20 (34)
Currently drinking alcohol	566 (51)	333 (49)	114 (48)	32 (54)
APRI	0.63 (0.38–1.24)	0.52 (0.36–0.78)	0.52 (0.36–0.81)	0.70 (0.47–0.97)
IFNL genotype rs8099917 TT	596 (65)^a^	333 (60)^b^	127 (60)	41 (70)
Receiving HIV therapy	903 (81)	538 (79)	191 (81)	46 (78)
Undetectable HIV viral load, (<50 copies/ml)	682 (61)	395 (59)	135 (59)	37 (65)
CD4 count, cells/μl	380 (249–550)	400 (270–568)	380 (250–540)	377 (230–540)
HCV duration, years	18 (11–26)	18 (10–25)	18 (11–26)	18 (12–24)
HCV genotype 3	166 (19)^c^	87 (16)^d^	30 (16)^e^	13 (26)^f^

**Abbreviations:** CCC, Canadian Co-infection Cohort; APRI, aspartate aminotransferase (AST) to platelet ratio index, calculated as follows: [(AST/upper limit of normal)/platelet count (109 /L)] x 100; IFNL, Interferon Lambda; HCV, Hepatitis C virus. Presented as n(%) or Median (Interquartile Range).

a. IFNL genotype available in 917 individuals

b. IFNL genotype available in 551 individuals

c. HCV genotype data available in only 874 individuals

d. HCV genotype data available in only 549 individuals

e. HCV genotype data available in only 189 individuals

f. HCV genotype data available in only 50 individuals

**Table 2:** Multivariable results of Cox models analyzing the association of significant liver fibrosis [HR (95% CI)] before and after multiple imputation.

COX MODEL RESULTS [HR (95% CI)]	Before imputation		After imputation	
Included Predictors^a^	Model 1	Model 2	Model 1	Model 2
Female	1.11 (0.64, 1.94)	1.34 (0.54, 3.34)	1.25 (0.82, 1.90)	1.35 (0.82, 2.21)
Current alcohol use	1.25 (0.73, 2.12)	0.90 (0.46, 1.75)	1.31 (0.89, 1.92)	1.30 (0.85, 2.00)
HIV viral load ^i^	1.43 (0.84, 2.44)	1.51 (0.78, 2.95)	1.17 (0.79, 1.74)	1.21 (0.80, 1.85)
Log Baseline APRI ^ii^	3.43 (1.92, 6.12)	2.91 (1.54, 5.50)	3.19 (2.05, 4.96)	2.71 (1.72, 4.26)
Age	0.99 (0.92, 1.06)	1.00 (0.91, 1.11)	1.00 (0.95, 1.05)	0.99 (0.95, 1.04)
Age*	0.98 (0.91, 1.05)	0.94 (0.86, 1.04)	0.99 (0.93, 1.04)	0.99 (0.93, 1.05)
HCV genotype 3^iii^	1.37 (0.73, 2.57)	1.04 (0.44, 2.48)	1.34 (0.80, 2.25)	1.36 (0.79, 2.36)
rs8099917 TT ^iv^	—	2.12 (1.01, 4.46)	—	1.39 (0.90, 2.16)
IL-8 ^v^	—	2.09 (1.44, 3.04)	—	1.48 (1.08, 2.02)
sICAM-1 ^vi^	—	3.85 (1.70, 8.75)	—	2.04 (1.05, 3.97)
RANTES ^vii^	—	0.58 (0.38, 0.88)	—	0.83 (0.64, 1.07)
hsCRP ^viii^	—	0.95 (0.74, 1.23)	—	0.95 (0.78, 1.16)
sCD14 ^ix^	—	0.36 (0.11, 1.19)	—	0.56 (0.24, 1.30)

a. Included immune markers (IL-8, sICAM-1, RANTES, and hsCRP, and sCD14) are log-transformed and centered.

* Restricted cubic spline function in age

i. Missing in 3% cases and noncases

ii. Missing in 6% of cases and 5% of noncases

iii. Missing in 14% of cases and 22% of noncases

iv. Missing in 1% of cases and 13% of noncases

v. Missing in 24% of cases and 28% of noncases

vi. Missing in 24% of cases and 28% of noncases

vii. Missing in 24% of cases and 28% of noncases

viii. Missing in 24% of cases and 30% of noncases

ix. Missing in 24% of cases and 28% of noncases

**Abbreviations:** HR, hazard ratio; CI, confidence interval; APRI, aspartate aminotransferase (AST) to platelet ratio index, calculated as follows: [(AST/upper limit of normal)/platelet count (109 /L)] x 100; HCV, Hepatitis C virus; IL-8, interleukin-8; sICAM-1, soluble intercellular adhesion molecule 1; RANTES, Regulated upon Activation, Normal T cell Expressed and Secreted protein; hsCRP high-sensitivity C-reactive protein; sCD14, soluble CD14.

One hundred and thirteen individuals developed significant liver fibrosis over 1300 years of risk for an event rate of 8.63 per 100 person-years (95% CI: 7.08, 10.60 per 100 py). Significant liver fibrosis cases were much more likely to be female, currently drinking alcohol, infected with HCV genotype 3 and carriers of the rs8099917 TT genotype (Table 2). Surprisingly, they were also more likely to have undetectable levels of HIV RNA at first visit. As expected, even at baseline, the median APRI value was higher among cases than those in the subcohort.

Immune marker values were available for 74% of the individuals in the analytic sample. The median values of the following immune markers were higher in cases than the subcohort: IL-8, MIP1α, MIP1β, TNFα, CXCL9, sICAM-1 and sVCAM-1 (S2 Table, S1 Fig). The remaining markers (RANTES, TGF-β1, MCP-1, CXCL11, sCD14 and hs-CRP) showed the opposite, that is, were lower in cases than the subcohort. The genetic marker at IFNL rs8099917 was available in 92% of the individuals included in the analysis. The pro-inflammatory TT genotype was more common in cases than the subcohort (Table 2).

The final clinical predictors in Model 1 were sex, current alcohol use (yes or no), HIV viral load (undetectable or not at ≤ 50 copies), natural log-transformed baseline APRI, HCV genotype 3 and age (Table 2). Other factors like CD4 count or ethnicity did not improve predictive ability and therefore were not included. Model 1 was nested in Model 2, which also included the genetic marker IFNL rs8099917 and the following 5 log-transformed immune markers: IL-8, sICAM-1, RANTES, hsCRP, and sCD14 (Table 2). While other combinations of markers and predictors were tested, these markers were selected because they target different stages of liver fibrosis development, are known risk factors for fibrosis, improved model fit or discrimination or displayed the strongest associations with the outcome in univariable analyses (S3 Table). Even in multivariable analysis, most of the selected markers had a stronger association with the outcome than any of the clinical predictors with the exception of baseline APRI (Table 3). The selected markers were also strongly correlated with the other markers that were not included.

**Table 3:** Predictive accuracy for 3-year risk of significant liver fibrosis using model 1 (clinical predictors only) and model 2 (Model 1+ Selected Markers).

PREDICTIVE ACCURACY	Before imputation		After imputation	
	Model 1	Model 2	Model 1	Model 2
**Discrimination:**				
Harrell’s C-index (95% CI)	0.731 (0.647, 0.815)	0.819 (0.740, 0.899)	0.730 (0.670, 0.789)	0.762 (0.703, 0.820)
**Calibration (p-values)**^**a**^				
Hosmer-Lemeshow	0.32	0.30	0.37	0.47
Gronnesby and Borgan (GB) test	0.76	0.59	0.47	0.88

a. Using quintiles of risk. Results similar with tertiles, as the number and threshold of cutpoints can affect statistical tests.

Model 1 included the following clinical predictors: sex, current alcohol use, HIV viral load, baseline APRI, HCV genotype 3 and age. Model 2 included Model 1 predictors and the following: genetic marker at IFNL rs8099917 and 5 immune markers IL-8, sICAM-1, RANTES, hsCRP, and sCD14.

The bootstrapped Harrell’s C indexes differed between models 1 and 2, regardless of multiple imputation (Table 2). The higher values for model 2 versus model 1 indicated that adding the six markers improved the discrimination capacity beyond that of traditional clinical factors. Using only clinical risk factors indicated that there was a 73% probability that predicted risk is higher for cases than non-cases. That probability rose to 76% using selected markers. Other clinical factors such as CD4 count or the remaining immune markers did not substantially increase the C-index or improve model fit as measured by the AIC. Variables included in indexes from other studies such as hyaluronic acid, total bilirubin, or GGT also did not increase the C-index, improve calibration, or improve model fit.

Both models 1 and 2 were well-calibrated as measured by the Hosmer-Lemeshow test and the GB test (p>0.05 in both tests, Table 2 and S2 Fig). This indicated that there were no significant deviations by observed risk from subgroups of predicted risk estimates, regardless of number or location of cutpoints. Risk stratification also appeared to improve with the inclusion of the genetic and immune markers in model 2. The category-based NRI indicated that the classification improved more in those not developing fibrosis (i.e. the non-events) than in those with the outcome: 5.1% were correctly reclassified (i.e. moved to a higher risk category) in those with events, while almost 21% of the non-events were correctly recategorized (i.e. correctly moved to a lower risk category) (Table 4). The continuous NRI, on the other hand, demonstrated improvement in both, with a greater movement among cases than non-cases (Table 5).

**Table 4:** Net reclassification improvement (NRI) index for 3-year risk of significant liver fibrosis using model 1 (clinical predictors only) and model 2 (Model 1+ Selected Markers).

		MODEL 2				Reclassified						
	MODEL 1	< = 10	>10–25	>25	Total	N	Higher		Lower		Net	NRI
Developed fibrosis**	< = 10	2	**0**	**1**	3	150						
	>10–25	4	6	**11**	21		**12**	**20.3%**	9	15.2%	5.1	
	>25	1	4	30	35							
	Total	7	10	42	59**							26
											
**No fibrosis**	< = 10	16	**5**	**2**	23							
	>10–25	24	10	**11**	45		**18**	**19.8%**	37	40.6%	20.9	
	>25	4	9	10	23							
	Total	44	24	23	**91**							

Gray = movement to a lower risk category; Orange = movement to a higher risk category

**Without multiple imputation, only 59 cases had event times within 3 years and had complete information to be used in the analysis. Results were similar if multiple imputation was used to complete information for 94 cases (not shown).

Model 1 included the following clinical predictors: sex, current alcohol use, HIV viral load, baseline APRI, HCV genotype 3 and age. Model 2 included Model 1 predictors and the following: genetic marker at IFNL rs8099917 and 5 immune markers IL-8, sICAM-1, RANTES, hsCRP, and sCD14.

**Table 5:** Comparison of category-based and continuous net reclassification improvement (NRI) indexes for 3-year risk of significant liver fibrosis using model 1 (clinical predictors only) and model 2 (Model 1+ Selected Markers).

	Category-based NRI (p-value)	Continuous NRI (p-value)
Events	0.051 (0.51)	0.356 (0.006)
Nonevents	0.209 (0.010)	0.187 (0.075)
**Overall**	0.26 (0.02)	0.543 (0.001)

NRI can be defined as the sum of improvements in risk classification in events and non-events. It is measured separately in those with the outcome and those without the outcome—the sum of differences in proportions of individuals moving up minus the proportion moving down for those with the outcome, and the proportion of individuals moving down minus the proportion moving up for those without the outcome.
$${NRI}_{events}=\frac{Number\;of\;events\;moving\;to\;higher\;risk\;category-\;Number\;of\;events\;moving\;to\;lower\;risk\;category}{Total\;number\;of\;events}$$
$${NRI}_{nonevents}=\frac{Number\;of\;nonevents\;moving\;to\;lower\;risk\;category-\;Number\;of\;nonevents\;moving\;to\;higher\;risk\;category}{Total\;number\;of\;nonevents}$$

NRI_overall_ = NRI_events_ + NRI_nonevents_

## Discussion

Our results demonstrate that specific immune markers improved ability to predict 3-year risk of significant liver fibrosis over traditional clinical factors alone in a cohort representative of HIV-HCV co-infected Canadians. While improvement in discrimination was modest, adding immune markers improved risk classification. Since this is a population at high risk for accelerated liver fibrosis, there is a great need for better clinical prognosis and risk assessment; such a prognostic tool may facilitate prioritizing expensive HCV treatment by improving identification of those for whom it may be safely delayed.

We tested clinical predictors linked with fibrosis that were most likely to be available to clinicians. The immune markers we screened, however, are not routinely collected at a clinical setting and are not cheap to measure (for the 6 additional markers included in Model 2, costs were approximately $110 for reagents alone). Nevertheless, the blood draw required for immune marker measures is less invasive than a biopsy and does not require specialist appointments or equipment and is far less expensive than current HCV treatment. Many of the markers were correlated with hepatic levels[45] and with each other, so we selected those most representative of different stages of fibrosis development, those with the strongest links to fibrosis, and those most likely to improve discrimination.

Other than baseline APRI, many of the selected markers displayed stronger relationships with fibrosis than any clinical predictor studied. In univariable and multivariable analysis, both IL-8 and sICAM-1 acted profibrogenically, being linked with a higher rate of significant fibrosis. These markers also appeared to behave in a dose-dependent manner when examined at the median and quartile level. RANTES and hs-CRP, on the other hand, appeared protective in univariable analysis (S3). We summarized other studies that could account for these trends from an etiological perspective (S1 Table). However, as our purpose in modeling them was solely for prediction, we cannot infer any causality from these associations.

Other factors, such as hyaluronic acid [22, 34], total bilirubin [21, 22], or GGT [21–23] have been included in other fibrotic indexes but did not improve model fit or prognostic ability in our study. In studies of the Hepascore and the SHASTA index [22, 34], these variables were collected not for predicting risk but for diagnostic and staging purposes in individuals who had already developed liver disease. As such, they were collected at the same time point as the liver biopsy sample, which was used to determine the outcome (fibrosis or cirrhosis). In our study, however, the markers were measured in disease-free individuals up to three years before significant fibrosis set in, thus somewhat incorporating the stochastic nature of prognosis[46].

**Fig 2:**
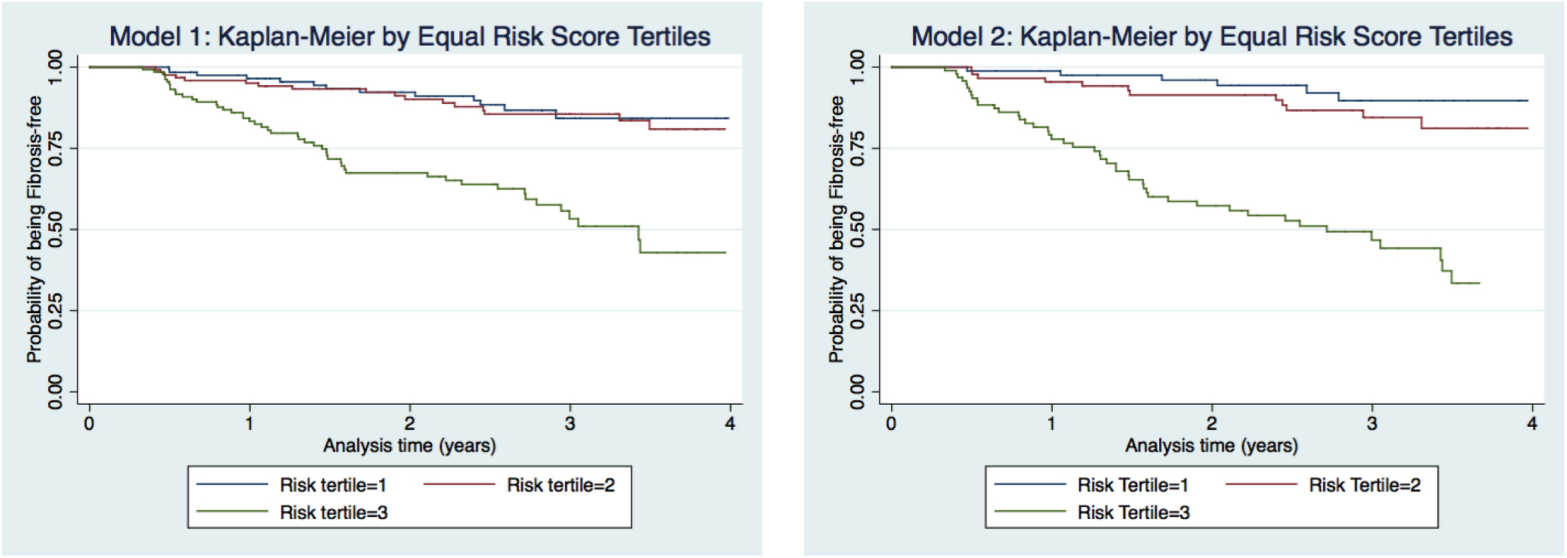
Tertiles of risk score against Kaplan-Meier estimates of risk of significant liver fibrosis. Blue = score tertile 1 (lowest risk); Red = score tertile 2; Green = score tertile 3 (highest risk). Model 1 included the following clinical predictors: sex, current alcohol use, HIV viral load, baseline APRI, HCV genotype 3 and ageModel 2 included Model 1 predictors and the following: genetic marker at IFNL rs8099917 and 5 immune markers IL-8, sICAM-1, RANTES, hsCRP, and sCD14 The wider separation of the Kaplan-Meier curves in Model 2 provides a visual representation of the improvement in discrimination with the addition of the markers compared to Model 1. The risk score is constructed from the linear predictors of the Cox model. The linear predictor is a weighted sum of the variables in the final model, where the weights are the regression coefficients. (See also S4 Table).

Using these chosen six markers improved our ability to discriminate between those who develop significant liver fibrosis and those who do not, as indicated by the higher C-indexes in model 2 versus model 1 and the wider separation of the Kaplan-Meier curves from the risk score tertiles (Fig 2), though the improvement was minor and not statistically significant. Nevertheless, discrimination by both models was similar to other comparable indexes such as the prognostic score for prediction of ESLD in HIV-HCV co-infected individuals on cART (C-statistic = 0.73), which can be considered good and of some clinical utility [47]. Other prognostic indexes which predicted risk of mortality in cirrhotics (Child-Pugh score, MELD score) or in HIV-infected individuals on cART (VACS Index) [48] had C-statistics around 0.80 or higher, which are considered very good or excellent [49, 50].

While improvement in discrimination was modest, inclusion of immune markers improved the net reclassification improvement index, as indicated by both category-based and continuous NRI estimates. Results from the category-based NRI indicate that measuring the markers in model 2 correctly reduced risk estimates in those who did not develop liver fibrosis. While this does not enable identification of higher-risk individuals for treatment [51], it could help identify patients for whom treatment might be safely deferred (up to 21% of individuals who eventually did not develop fibrosis over 3 years). The continuous NRI, on the other hand, indicated that model 2 improved ability to identify higher-risk individuals for treatment. However, these changes in predicted risk might not be clinically significant, as values of continuous NRIs are often higher than their category-based counterparts [51]. When viewed together with the Harrell’s C-index, the NRI seemed to support improved discrimination and risk classification with inclusion of genetic and inflammation markers. These results, however, must be interpreted with caution. Category-based NRIs are sensitive to the number of risk categories as well as the chosen cutpoints [42], while continuous NRIs can have large values for even weak markers [51]. A cost-benefit analysis that takes into account the expense of these markers as well as the high cost of HCV treatment might be useful as a next step.

The strength of our study includes a large source population that is broadly representative of Canadian co-infected patients, thus making our results directly generalizable and clinically relevant. This is important as marker and disease prevalence or marker correlations with other known risk factors can all affect the estimates of the discriminatory capacity of a marker [52]. Inclusion of specific markers in model 2 appeared to provide higher discrimination than the clinical predictors in model 1 whether multiple imputation was used or not. Finally, the list of immune markers we tested target different stages of the underlying mechanism of fibrosis progression, enhancing our ability to capture the outcome at various stages of development.

The limitations of our study include having marker measures at only visit 1 or 2, so we are unable to assess the predictive value of markers at other time points or measure the predictive value of changes of marker levels. However, as we were interested in prognosis, using the first available sample mirrors what would occur in clinical practice when a patient is first evaluated. We also lacked the power to assess interaction or effect measure modification of markers in different subgroups. Furthermore, missing samples in the subcohort and cases also reduced power, which we addressed with multiple imputation. Weakened associations after imputation can be caused by misspecification of the imputation model or if variables capturing the missing mechanism are excluded [53]. However, our results did not change with different imputation models. Since our primary goal was to assess the prognostic ability of specific markers, using the full sample size gave us sufficient power to assess calibration and discrimination properly, despite some potential misspecification. HCV duration, while informative, is hard to estimate in a real-life clinical scenario and is only approximate in our study. We chose to use age instead to capture some of the time element that HCV duration would have provided. Most of the injection drug users in our study first started injecting around the same age, approximately 18 years before cohort entry. Our study modeled the markers specifically for prognostic assessment precluding causal inferences. Finally, external validation in an independent dataset should be performed before applying this model clinically. Data provided in S2 and S4 Tables will be especially relevant to future external validation studies.

In conclusion, we found that in an HIV-HCV co-infected population, incorporating a genetic marker from IFNL rs8099917 and the immune markers IL-8, sICAM-1, RANTES, hs-CRP, and sCD14 allowed us to better predict the 3-year risk of significant liver fibrosis over traditional clinical risk factors alone. While the improvement in discrimination was small, the model with the markers also classified risk and fit better than the one without the markers. To assess whether this improvement justifies the additional cost of measuring these markers in the face of highly expensive HCV treatment requires further cost-benefit analyses.

## Supporting information

S1 Table Summary of immune markers of interest. (DOC)

S2 Table Distribution of immune markers in random subcohort and cases outside subcohort. (DOC)

S3 Table Univariable results of the association of significant liver fibrosis with log-transformed immune markers [HR (95% CI)]. (DOC)

S4 Table Estimated regression coefficients and standard errors for calculating risk score [estimated beta (SE)] in final models 1 and 2. (DOC)

S1 Fig Medians of log-transformed immune markers: Subcohort vs. cases outside subcohort. Abbreviations: TGF-β1, transforming growth factor beta 1; sICAM-1, soluble intercellular adhesion molecule 1; sVCAM-1, soluble vascular cell adhesion molecule 1; RANTES, Regulated upon Activation, Normal T cell Expressed and Secreted protein; sCD14, soluble CD14; TNF-α, tumor necrosis factor alpha; MIP1β, macrophage inflammatory protein 1 beta; MCP-1, monocyte chemotactic protein-1; CXCL11, chemokine (C-X-C motif) ligand 11; CXCL9, chemokine (C-X-C motif) ligand 9; hsCRP high-sensitivity C-reactive protein; MIP1α, macrophage inflammatory protein 1 alpha; IL-8, interleukin-8.
(TIF)

S2 Fig Calibration with predicted survival curves and Kaplan-Meier estimates in model 1 (clinical predictors only) and Model 2 (Model 1 plus IFNL rs8099917 and 5 Selected Immune Markers). a)Left panel: Equal-sized Quintiles of 3-year Risk in Model 1 (top) vs. Model 2 (bottom)
Smooth lines represent predicted survival probabilities, and vertical capped lines denote Kaplan–Meier estimates with 95% confidence intervals. Five prognosis groups are plotted: the “Good” group (darkest lines) and the “Poor” group (palest lines) at the highest and lowest risk categories, respectively, with the other 3 in between.
b)Right panel: 3 Unequal Risk Groups (Cut at the 25^th^ and the 75^th^ Percentiles of the Failure Times)
Smooth lines represent predicted survival probabilities, and vertical capped lines denote Kaplan–Meier estimates with 95% confidence intervals. Three prognosis groups are plotted: the “Good” group (darkest lines), the “Intermediate” group (medium-dark lines), and the “Poor” group (paler lines). Using unequal sized risk groups allows identification of individuals with the most extreme prognosis [41].
Model 1 included the following clinical predictors: sex, current alcohol use, HIV viral load, baseline APRI, HCV genotype 3 and ageModel 2 included Model 1 predictors and the following: genetic marker at IFNL rs8099917 and 5 immune markers IL-8, sICAM-1, RANTES, hsCRP, and sCD14.
Abbreviations: IL-8, interleukin-8; sICAM-1, soluble intercellular adhesion molecule 1; RANTES, Regulated upon Activation, Normal T cell Expressed and Secreted protein; sCD14, soluble CD14; hsCRP high-sensitivity C-reactive protein.
(TIF)
