# Supplementary material for: A prognostic model for development of significant liver fibrosis in HIV-hepatitis C co-infection
Source: PLoS One. 2017 May 3;12(5):e0176282. doi: 10.1371/journal.pone.0176282 (PMC5415136; doi:10.1371/journal.pone.0176282)
Supplement: S1 Table — (DOC) [file pone.0176282.s001.doc]

**S1 Table. Summary of Immune Markers of Interest**

|  |  | **Description** | **References** |
| --- | --- | --- | --- |
| **Cytokines** | **TGF-β1** | - Triggered directly by HIV proteins - Called the most pro-fibrotic cytokine: directly stimulates molecules that are imbalanced in fibrosis | [1-4] |
|  | **TNF-α** | - Elevated in livers of co-infected individuals - Produced by liver macrophages in response to viruses, alcohol, and LPS - Upregulates HIV replication | [5-8] |
| **Chemokines** | **IL-8** | - Serum levels linked with HCV disease progression and interferon unresponsiveness - In cell cultures, HCV and HIV proteins both triggered IL-8 levels in a dose-dependent manner | [9-12] |
|  | **MCP-1** | - Not expressed in healthy livers but triggered by HCV infection - Stimulates fibrogenesis | [13, 14] |
|  | **MIP1α** | - Binds CCR5, receptor needed for HIV entry - CCR5-MIP1α pairs dominate during HCV infection - Levels higher in co-infected livers than mono-infected | [15]  [14] |
|  | **MIP1β** |  |  |
|  | **RANTES** | - Binds CCR5 - Stimulates fibrogenesis - Higher in co-infected livers vs. monoinfected | [13-17] |
|  | **CXCL9** | - Correlated to histologic liver disease activity after HCV infection - Significantly elevated in plasma in those with advanced liver necroinflammation | [18-20] |
|  | **CXCL11** | - Has chemoattractant properties that initiate and perpetuate liver inflammation-guides T cells through inflamed liver - Correlated to histologic disease activity in the liver after HCV infection | [18-21] |
| **Endothelial activation markers** | **sICAM-1** | - Higher in HIV-infected individuals - Associated with higher HIV viral load and faster HIV disease progression - Higher in cirrhotic patients | [22, 23] |
|  | **sVCAM-1** |  |  |
|  | **hsCRP** | - Produced in the liver - Released during the acute phase of an infection - Reproducible, dynamic reflection of ongoing tissue injury - In HIV-positive individuals, higher levels linked with higher risk of opportunistic infections and all-cause mortality - Trends unclear with HIV-HCV co-infection | [24-28] |
|  | **sCD14** | - Acts as a marker of microbial translocation - Co-receptor to LPS, a component of bacterial cell walls - Release in soluble form triggers immune activation and cytokine production - In HIV-infected, highest sCD14 levels linked with 6-fold higher risk of mortality vs. lowest levels of sCD14   - even after adjusting for other inflammatory markers, CD4 count and HIV viral load - In HCV mono and co-infection, higher levels linked with lower treatment response and cirrhosis - In co-infected individuals, highest quartile linked with 8-fold higher odds of cirrhosis vs. lowest quartile | [29-34] |

**^Abbreviations^:** TGF-β1, transforming growth factor beta 1; TNF-α, tumor necrosis factor alpha; IL-8, interleukin-8; LPS, lipopolysaccharide; MCP-1, monocyte chemotactic protein-1; MIP1α, macrophage inflammatory protein 1 alpha; MIP1β, macrophage inflammatory protein 1 beta; RANTES, Regulated upon Activation, Normal T cell Expressed and Secreted protein; CXCL9, chemokine (C-X-C motif) ligand 9; CXCL11, chemokine (C-X-C motif) ligand 11; sICAM-1, soluble intercellular adhesion molecule 1; sVCAM-1, soluble vascular cell adhesion molecule 1; hsCRP high-sensitivity C-reactive protein; sCD14, soluble CD14.

**REFERENCES**

1. Munshi N, Balasubramanian A, Koziel M, Ganju RK, Groopman JE. Hepatitis C and human immunodeficiency virus envelope proteins cooperatively induce hepatocytic apoptosis via an innocent bystander mechanism. The Journal of infectious diseases. 2003;188(8):1192-204. Epub 2003/10/11. doi: 10.1086/378643. PubMed PMID: 14551890.

2. Poggi A, Zocchi MR. HIV-1 Tat triggers TGF-beta production and NK cell apoptosis that is prevented by pertussis toxin B. Clinical & developmental immunology. 2006;13(2-4):369-72. Epub 2006/12/13. doi: 10.1080/17402520600645712. PubMed PMID: 17162379; PubMed Central PMCID: PMC2270756.

3. Bataller R, Paik YH, Lindquist JN, Lemasters JJ, Brenner DA. Hepatitis C virus core and nonstructural proteins induce fibrogenic effects in hepatic stellate cells. Gastroenterology. 2004;126(2):529-40. Epub 2004/02/06. PubMed PMID: 14762790.

4. Shek FW, Benyon RC. How can transforming growth factor beta be targeted usefully to combat liver fibrosis? European journal of gastroenterology & hepatology. 2004;16(2):123-6. Epub 2004/04/13. PubMed PMID: 15075983.

5. Poli G, Bressler P, Kinter A, Duh E, Timmer WC, Rabson A, et al. Interleukin 6 induces human immunodeficiency virus expression in infected monocytic cells alone and in synergy with tumor necrosis factor alpha by transcriptional and post-transcriptional mechanisms. The Journal of experimental medicine. 1990;172(1):151-8. Epub 1990/07/01. PubMed PMID: 2193094; PubMed Central PMCID: PMC2188185.

6. Poli G, Kinter A, Justement JS, Kehrl JH, Bressler P, Stanley S, et al. Tumor necrosis factor alpha functions in an autocrine manner in the induction of human immunodeficiency virus expression. Proceedings of the National Academy of Sciences of the United States of America. 1990;87(2):782-5. Epub 1990/01/01. PubMed PMID: 2300561; PubMed Central PMCID: PMC53350.

7. Folks TM, Clouse KA, Justement J, Rabson A, Duh E, Kehrl JH, et al. Tumor necrosis factor alpha induces expression of human immunodeficiency virus in a chronically infected T-cell clone. Proceedings of the National Academy of Sciences of the United States of America. 1989;86(7):2365-8. Epub 1989/04/01. PubMed PMID: 2784570; PubMed Central PMCID: PMC286913.

8. Decrion AZ, Dichamp I, Varin A, Herbein G. HIV and inflammation. Current HIV research. 2005;3(3):243-59. Epub 2005/07/19. PubMed PMID: 16022656.

9. Polyak SJ, Khabar KS, Rezeiq M, Gretch DR. Elevated levels of interleukin-8 in serum are associated with hepatitis C virus infection and resistance to interferon therapy. Journal of virology. 2001;75(13):6209-11. Epub 2001/06/08. doi: 10.1128/JVI.75.13.6209-6211.2001. PubMed PMID: 11390624; PubMed Central PMCID: PMC114338.

10. Zimmermann HW, Seidler S, Gassler N, Nattermann J, Luedde T, Trautwein C, et al. Interleukin-8 is activated in patients with chronic liver diseases and associated with hepatic macrophage accumulation in human liver fibrosis. PloS one. 2011;6(6):e21381. Epub 2011/07/07. doi: 10.1371/journal.pone.0021381. PubMed PMID: 21731723; PubMed Central PMCID: PMC3120868.

11. Clement S, Pascarella S, Conzelmann S, Gonelle-Gispert C, Guilloux K, Negro F. The hepatitis C virus core protein indirectly induces alpha-smooth muscle actin expression in hepatic stellate cells via interleukin-8. Journal of hepatology. 2010;52(5):635-43. Epub 2010/03/30. doi: 10.1016/j.jhep.2009.10.035. PubMed PMID: 20347177.

12. Balasubramanian A, Ganju RK, Groopman JE. Hepatitis C virus and HIV envelope proteins collaboratively mediate interleukin-8 secretion through activation of p38 MAP kinase and SHP2 in hepatocytes. The Journal of biological chemistry. 2003;278(37):35755-66. Epub 2003/06/26. doi: 10.1074/jbc.M302889200. PubMed PMID: 12824191.

13. Schwabe RF, Bataller R, Brenner DA. Human hepatic stellate cells express CCR5 and RANTES to induce proliferation and migration. American journal of physiology Gastrointestinal and liver physiology. 2003;285(5):G949-58. Epub 2003/06/28. doi: 10.1152/ajpgi.00215.2003. PubMed PMID: 12829440.

14. Reeves HL, Friedman SL. Activation of hepatic stellate cells--a key issue in liver fibrosis. Frontiers in bioscience : a journal and virtual library. 2002;7:d808-26. Epub 2002/03/19. PubMed PMID: 11897564.

15. Kuntzen T, Tural C, Li B, Feldmann G, Kupfer B, Nischalke HD, et al. Intrahepatic mRNA expression in hepatitis C virus and HIV/hepatitis C virus co-infection: infiltrating cells, cytokines, and influence of HAART. AIDS. 2008;22(2):203-10. Epub 2007/12/22. doi: 10.1097/QAD.0b013e3282f3553b. PubMed PMID: 18097222.

16. Berres ML, Koenen RR, Rueland A, Zaldivar MM, Heinrichs D, Sahin H, et al. Antagonism of the chemokine Ccl5 ameliorates experimental liver fibrosis in mice. The Journal of clinical investigation. 2010;120(11):4129-40. doi: 10.1172/JCI41732. PubMed PMID: 20978355; PubMed Central PMCID: PMCPMC2964968.

17. Gonzalez EO, Boix V, Deltoro MG, Aldeguer JL, Portilla J, Montero M, et al. The effects of Maraviroc on liver fibrosis in HIV/HCV co-infected patients. J Int AIDS Soc. 2014;17(4 Suppl 3):19643. doi: 10.7448/IAS.17.4.19643. PubMed PMID: 25394147; PubMed Central PMCID: PMCPMC4224825.

18. Moura AS, Carmo RA, Teixeira AL, Leite VH, Rocha MO. Soluble inflammatory markers as predictors of liver histological changes in patients with chronic hepatitis C virus infection. European journal of clinical microbiology & infectious diseases : official publication of the European Society of Clinical Microbiology. 2010;29(9):1153-61. Epub 2010/06/19. doi: 10.1007/s10096-010-0981-4. PubMed PMID: 20559676.

19. Zeremski M, Dimova R, Brown Q, Jacobson IM, Markatou M, Talal AH. Peripheral CXCR3-Associated Chemokines as Biomarkers of Fibrosis in Chronic Hepatitis C Virus Infection. Journal of Infectious Diseases. 2009;200(11):1774-80. doi: 10.1086/646614.

20. Zeremski M, Petrovic LM, Talal AH. The role of chemokines as inflammatory mediators in chronic hepatitis C virus infection. Journal of viral hepatitis. 2007;14(10):675-87. Epub 2007/09/19. doi: 10.1111/j.1365-2893.2006.00838.x. PubMed PMID: 17875002.

21. Helbig KJ, Ruszkiewicz A, Semendric L, Harley HA, McColl SR, Beard MR. Expression of the CXCR3 ligand I‐TAC by hepatocytes in chronic hepatitis C and its correlation with hepatic inflammation. Hepatology. 2004;39(5):1220-9.

22. Giron-Gonzalez JA, Martinez-Sierra C, Rodriguez-Ramos C, Rendon P, Macias MA, Fernandez-Gutierrez C, et al. Adhesion molecules as a prognostic marker of liver cirrhosis. Scandinavian journal of gastroenterology. 2005;40(2):217-24. Epub 2005/03/15. doi: 10.1080/00365520510011470. PubMed PMID: 15764154.

23. Wolf K, Tsakiris DA, Weber R, Erb P, Battegay M. Antiretroviral therapy reduces markers of endothelial and coagulation activation in patients infected with human immunodeficiency virus type 1. The Journal of infectious diseases. 2002;185(4):456-62. Epub 2002/02/28. doi: 10.1086/338572. PubMed PMID: 11865397.

24. Lau B, Sharrett AR, Kingsley LA, Post W, Palella FJ, Visscher B, et al. C-reactive protein is a marker for human immunodeficiency virus disease progression. Archives of internal medicine. 2006;166(1):64-70. Epub 2006/01/13. doi: 10.1001/archinte.166.1.64. PubMed PMID: 16401812.

25. Pinato DJ, Bains J, Irkulla S, Pomroy J, Ujam B, Gaze D, et al. Advanced age influences the dynamic changes in circulating C-reactive protein following injury. Journal of clinical pathology. 2013;66(8):695-9. doi: 10.1136/jclinpath-2012-201374. PubMed PMID: 23539737.

26. Kuller LH, Tracy R, Belloso W, De Wit S, Drummond F, Lane HC, et al. Inflammatory and coagulation biomarkers and mortality in patients with HIV infection. PLoS medicine. 2008;5(10):e203. Epub 2008/10/24. doi: 10.1371/journal.pmed.0050203. PubMed PMID: 18942885; PubMed Central PMCID: PMC2570418.

27. Rodger AJ, Fox Z, Lundgren JD, Kuller LH, Boesecke C, Gey D, et al. Activation and coagulation biomarkers are independent predictors of the development of opportunistic disease in patients with HIV infection. The Journal of infectious diseases. 2009;200(6):973-83. Epub 2009/08/15. doi: 10.1086/605447. PubMed PMID: 19678756; PubMed Central PMCID: PMC2892757.

28. Reingold J, Wanke C, Kotler D, Lewis C, Tracy R, Heymsfield S, et al. Association of HIV infection and HIV/HCV coinfection with C-reactive protein levels: the fat redistribution and metabolic change in HIV infection (FRAM) study. J Acquir Immune Defic Syndr. 2008;48(2):142-8. Epub 2008/03/18. doi: 10.1097/QAI.0b013e3181685727. PubMed PMID: 18344877; PubMed Central PMCID: PMC2561207.

29. Marchetti G, Tincati C, Silvestri G. Microbial translocation in the pathogenesis of HIV infection and AIDS. Clinical microbiology reviews. 2013;26(1):2-18. doi: 10.1128/CMR.00050-12. PubMed PMID: 23297256; PubMed Central PMCID: PMC3553668.

30. Caradonna L, Mastronardi ML, Magrone T, Cozzolongo R, Cuppone R, Manghisi OG, et al. Biological and clinical significance of endotoxemia in the course of hepatitis C virus infection. Current pharmaceutical design. 2002;8(11):995-1005. PubMed PMID: 11945146.

31. Marchetti G, Cozzi-Lepri A, Tincati C, Calcagno A, Ceccherini-Silberstein F, De Luca A, et al. Immune activation and microbial translocation in liver disease progression in HIV/hepatitis co-infected patients: results from the Icona Foundation study. BMC infectious diseases. 2014;14:79. doi: 10.1186/1471-2334-14-79. PubMed PMID: 24520976; PubMed Central PMCID: PMC3923986.

32. Shive CL, Jiang W, Anthony DD, Lederman MM. Soluble CD14 is a nonspecific marker of monocyte activation. AIDS. 2015;29(10):1263-5. doi: 10.1097/QAD.0000000000000735. PubMed PMID: 26035325; PubMed Central PMCID: PMC4452959.

33. Sandler NG, Wand H, Roque A, Law M, Nason MC, Nixon DE, et al. Plasma levels of soluble CD14 independently predict mortality in HIV infection. The Journal of infectious diseases. 2011;203(6):780-90. Epub 2011/01/22. doi: 10.1093/infdis/jiq118. PubMed PMID: 21252259; PubMed Central PMCID: PMC3071127.

34. Balagopal A, Philp FH, Astemborski J, Block TM, Mehta A, Long R, et al. Human immunodeficiency virus-related microbial translocation and progression of hepatitis C. Gastroenterology. 2008;135(1):226-33. Epub 2008/05/07. doi: 10.1053/j.gastro.2008.03.022. PubMed PMID: 18457674; PubMed Central PMCID: PMC2644903.
