# Supplementary material for: A prognostic model for development of significant liver fibrosis in HIV-hepatitis C co-infection
Source: PLoS One. 2017 May 3;12(5):e0176282. doi: 10.1371/journal.pone.0176282 (PMC5415136; doi:10.1371/journal.pone.0176282)
Supplement: S2 Table — (DOC) [file pone.0176282.s002.doc]

**S2 Table. Distribution of Immune Markers in Random Subcohort and Cases Outside Subcohort**

|  | **Subcohort, n=171** | **Cases outside subcohort, n=46** |
| --- | --- | --- |
| **IL-8** |  |  |
| **Average (SD)** | 11.87 (12.54) | 14.47 (12.7) |
| **Median (IQR)** | 8.58 (5.20-13.94) | 10.37 (7.51-16.92) |
| **sICAM-1** |  |  |
| **Average (SD)** | 214,405 (92,171) | 231,504 (89,213) |
| **Median (IQR)** | 189,927 (158,884-250,298) | 216,092 (182,731- 258,416) |
| **RANTES** |  |  |
| **Average (SD)** | 68,786.33 (52,971) | 57,956.3 (65,551) |
| **Median (IQR)** | 63,970 (25,527-97,741) | 33,058 (18,120-73,487) |
| **sCD14** |  |  |
| **Average (SD)** | 1.79 x10^6^ (571,015) | 1.76 x10^6^ (520,672) |
| **Median (IQR)** | 1.72 x10^6^ (1.44 x10^6^-2.03 x10^6^) | 1.69 x10^6^ (1.45 x10^6^- 2.11 x10^6^) |
| **hsCRP** |  |  |
| **Average (SD)** | 3.14 x10^6^ (5.77x10^6^) | 1.59 x10^6^ (1.84 x10^6^) |
| **Median (IQR)** | 1.29 x10^6^ (0.49 x10^6^-2.74 x10^6^) | 0.74 x10^6^ (0.29 x10^6^-2.10 x10^6^) |
| **MCP-1** |  |  |
| **Average (SD)** | 278.97 (179) | 267.80 (157) |
| **Median (IQR)** | 235 (150-344) | 223 (157-357) |
| **MIP1α** |  |  |
| **Average (SD)** | 2.78 (5.40) | 3.08 (3.62) |
| **Median (IQR)** | 0.39 (0.28-3.46) | 1.37 (0.28-5.41) |
| **MIP1β** |  |  |
| **Average (SD)** | 29.71 (25.25) | 38.13 (33.07) |
| **Median (IQR)** | 22.33 (13.46- 37.02) | 31.35(17.16-47.55) |
| **TNFα** |  |  |
| **Average (SD)** | 14.02 (9.15) | 15.30 (9.07) |
| **Median (IQR)** | 12.07 (7.63-17.73) | 20.19 (13.44- 24.53) |
| **sVCAM-1** |  |  |
| **Average (SD)** | 1,310,723 (434,816.8) | 1,382,531 (393,647) |
| **Median (IQR)** | 1.28 x10^6^ (1.02 x10^6^-1.51x10^6^) | 1.34 x10^6^ (1.10x10^6^-1.71x10^6^) |
| **CXCL9** |  |  |
| **Average (SD)** | 2875.94 (2303.19) | 3123 (2216.94) |
| **Median (IQR)** | 2265 (1444-3619) | 2337 (1567-4156) |
| **CXCL11** |  |  |
| **Average (SD)** | 274.50 (231) | 316.30 (279) |
| **Median (IQR)** | 210 (116-360) | 202 (134-425) |
| **TGF-β1** |  |  |
| **Average (SD)** | 21194.81 (15866) | 19,707.1 (20,503) |
| **Median (IQR)** | 17,385 (8731-29232) | 10,406 (5668-32,814) |

Units: all in pg/ml

**^Abbreviations^:** SD, standard deviation; IQR, interquartile range; IL-8, interleukin-8; sICAM-1, soluble intercellular adhesion molecule 1; RANTES, Regulated upon Activation, Normal T cell Expressed and Secreted protein; sCD14, soluble CD14; hsCRP high-sensitivity C-reactive protein; MCP-1, monocyte chemotactic protein-1; MIP1α, macrophage inflammatory protein 1 alpha; MIP1β, macrophage inflammatory protein 1 beta; TNF-α, tumor necrosis factor alpha; sVCAM-1, soluble vascular cell adhesion molecule 1; CXCL9, chemokine (C-X-C motif) ligand 9; CXCL11, chemokine (C-X-C motif) ligand 11; TGF-β1, transforming growth factor beta 1.
