# Supplementary material for: A prognostic model for development of significant liver fibrosis in HIV-hepatitis C co-infection
Source: PLoS One. 2017 May 3;12(5):e0176282. doi: 10.1371/journal.pone.0176282 (PMC5415136; doi:10.1371/journal.pone.0176282)
Supplement: S3 Table — (DOC) [file pone.0176282.s003.doc]

**S3 Table. Univariable Results of the Association of Significant Liver Fibrosis with Log-transformed Immune markers [HR (95% CI)]**

|  | **Not imputed** | **After Multiple Imputation** |
| --- | --- | --- |
| **IL-8** | 1.59 (1.20, 2.11) | 1.37 (1.08, 1.74) |
| **MCP-1** | 1.01 (0.71, 1.46) | 1.06 (0.75, 1.49) |
| **MIP1α** | 1.04 (0.91, 1.20) | 1.09 (0.93, 1.20) |
| **MIP1β** | 1.21 (0.88, 1.65) | 1.16 (0.89, 1.51) |
| **TNF-α** | 1.21 (0.84, 1.75) | 1.07 (0.76, 1.51) |
| **sICAM-1** | 3.22 (2.00, 5.18) | 2.66 (1.53, 4.61) |
| **sVCAM-1** | 2.38 (1.35, 4.20) | 1.88 (1.03, 3.41) |
| **RANTES** | 0.79 (0.63, 0.98) | 0.93 (0.76, 1.14) |
| **CXCL9** | 1.43 (1.08, 1.90) | 1.22 (0.91,1.64) |
| **CXCL11** | 1.30 (1.00, 1.70) | 1.26 (1.00, 1.59) |
| **TGF-β1** | 0.83 (0.64, 1.08) | 0.96 (0.76, 1.20) |
| **hsCRP** | 0.81 (0.68, 0.96) | 0.89 (0.76, 1.05) |
| **sCD14** | 1.14 (0.62, 2.08) | 1.08 (0.62, 1.88) |

**^Abbreviations^:** HR, hazard ratio; CI, confidence interval; IL-8, interleukin-8; MCP-1, monocyte chemotactic protein-1; MIP1α, macrophage inflammatory protein 1 alpha; MIP1β, macrophage inflammatory protein 1 beta; TNF-α, tumor necrosis factor alpha; sICAM-1, soluble intercellular adhesion molecule 1; sVCAM-1, soluble vascular cell adhesion molecule 1; RANTES, Regulated upon Activation, Normal T cell Expressed and Secreted protein; CXCL9, chemokine (C-X-C motif) ligand 9; CXCL11, chemokine (C-X-C motif) ligand 11; TGF-β1, transforming growth factor beta 1; hsCRP high-sensitivity C-reactive protein; sCD14, soluble CD14.
