# Supplementary material for: A prognostic model for development of significant liver fibrosis in HIV-hepatitis C co-infection
Source: PLoS One. 2017 May 3;12(5):e0176282. doi: 10.1371/journal.pone.0176282 (PMC5415136; doi:10.1371/journal.pone.0176282)
Supplement: S4 Table — (DOC) [file pone.0176282.s004.doc]

**S4 Table. Estimated Regression Coefficients and Standard Errors for Calculating Risk Score [Estimated beta (SE)] in Final Models 1 and 2**

|  | **Before imputation** | | **After imputation** | |
| --- | --- | --- | --- | --- |
|  | **Model 1** | **Model 2** | **Model 1** | **Model 2** |
| **Female** | 0.11 (0.28) | 0.29 (0.47) | 0.22 (0.21) | 0.30 (0.25) |
| **Current alcohol use** | 0.22 (0.27) | -0.10 (0.34) | 0.27 (0.20) | 0.26 (0.22) |
| **HIV viral load** | 0.36 (0.27) | 0.41 (0.34) | 0.16 (0.20) | 0.19 (0.21) |
| **Log Baseline APRI** | 1.23 (0.30) | 1.07 (0.32) | 1.16 (0.22) | 0.997 (0.23) |
| **Age** | -0.012 (0.04) | 0.003 (0.05) | -0.004 (0.02) | -0.007 (0.03) |
| **Age*** | -0.018 (0.04) | -0.058 (0.05) | -0.013 (0.03) | -0.01 (0.03) |
| **HCV genotype 3** | 0.32 (0.32) | 0.04 (0.44) | 0.29 (0.26) | 0.31 (0.28) |
| **rs8099917 TT** | -- | 0.75 (0.38) | -- | 0.33 (0.22) |
| **IL-8** | -- | 0.74 (0.19) | -- | 0.39 (0.16) |
| **sICAM-1** | -- | 1.35 (0.42) | -- | 0.71 (0.34) |
| **RANTES** | -- | -0.55 (0.21) | -- | -0.19 (0.13) |
| **hsCRP** | -- | -0.05 (0.13) | -- | -0.05 (0.11) |
| **sCD14** | -- | -1.02 (0.61) | -- | -0.58 (0.43) |

**^Abbreviations^:** APRI, aspartate aminotransferase (AST) to platelet ratio index, calculated as follows: [(AST/upper limit of normal)/platelet count (109 /L)] x 100; HCV, Hepatitis C virus; IL-8, interleukin-8; sICAM-1, soluble intercellular adhesion molecule 1; RANTES, Regulated upon Activation, Normal T cell Expressed and Secreted protein; hsCRP high-sensitivity C-reactive protein; sCD14, soluble CD14.

* Restricted cubic spline function in age

- Model 1 included the following clinical predictors: sex, current alcohol use, HIV viral load, baseline APRI, HCV genotype 3 and age.
- Model 2 included Model 1 predictors and the following: genetic marker at IFNL rs8099917 and 5 immune markers IL-8, sICAM-1, RANTES, hsCRP, and sCD14.
- In the final models, we used the natural log transformations of the continuous variables [immune markers (IL-8, sICAM-1, RANTES, hsCRP, and sCD14) and the baseline APRI] and centered at the mean.

The risk score is constructed from the linear predictors of the Cox model. The linear predictor is a weighted sum of the variables in the final model, where the weights are the regression coefficients. High values indicate a higher risk of significant liver fibrosis. The risk score for an individual is then the log relative hazard compared with a hypothetical individual whose risk score is zero [1]. In our dataset, an individual with a risk score zero is a 45-year-old male who is not currently drinking alcohol, with a detectable HIV viral load and host IFNL rs8099917 non-TT genotype, infected with HCV genotype 1, 2 or 4 and with mean values for APRI and the immune markers (IL-8, sICAM-1, RANTES, hsCRP, sCD14) at baseline.

**Reference**

1. Royston P, Altman DG. External validation of a Cox prognostic model: principles and methods. BMC Med Res Methodol. 2013;13:33. doi: 10.1186/1471-2288-13-33. PubMed PMID: 23496923; PubMed Central PMCID: PMC3667097.
