# Supplementary figures and images for: A prognostic model for development of significant liver fibrosis in HIV-hepatitis C co-infection
Source: PLoS One. 2017 May 3;12(5):e0176282. doi: 10.1371/journal.pone.0176282 (PMC5415136; doi:10.1371/journal.pone.0176282)

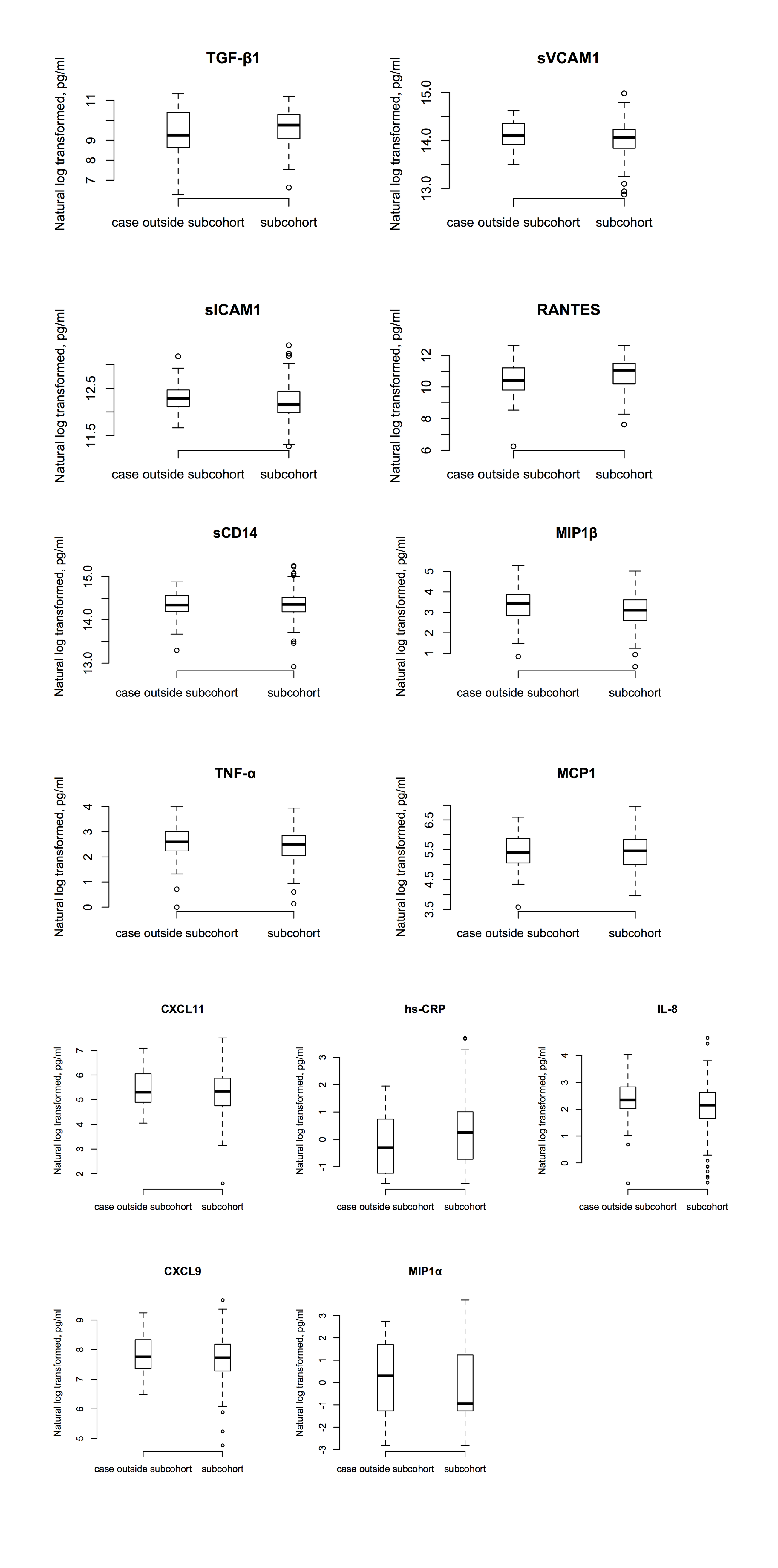

Supplement: S1 Fig — Abbreviations: TGF-β1, transforming growth factor beta 1; sICAM-1, soluble intercellular adhesion molecule 1; sVCAM-1, soluble vascular cell adhesion molecule 1; RANTES, Regulated upon Activation, Normal T cell Expressed and Secreted protein; sCD14, soluble CD14; TNF-α, tumor necrosis factor alpha; MIP1β, macrophage inflammatory protein 1 beta; MCP-1, monocyte chemotactic protein-1; CXCL11, chemokine (C-X-C motif) ligand 11; CXCL9, chemokine (C-X-C motif) ligand 9; hsCRP high-sensitivity C-reactive protein; MIP1α, macrophage inflammatory protein 1 alpha; IL-8, interleukin-8. (TIF) [file pone.0176282.s005.tif]

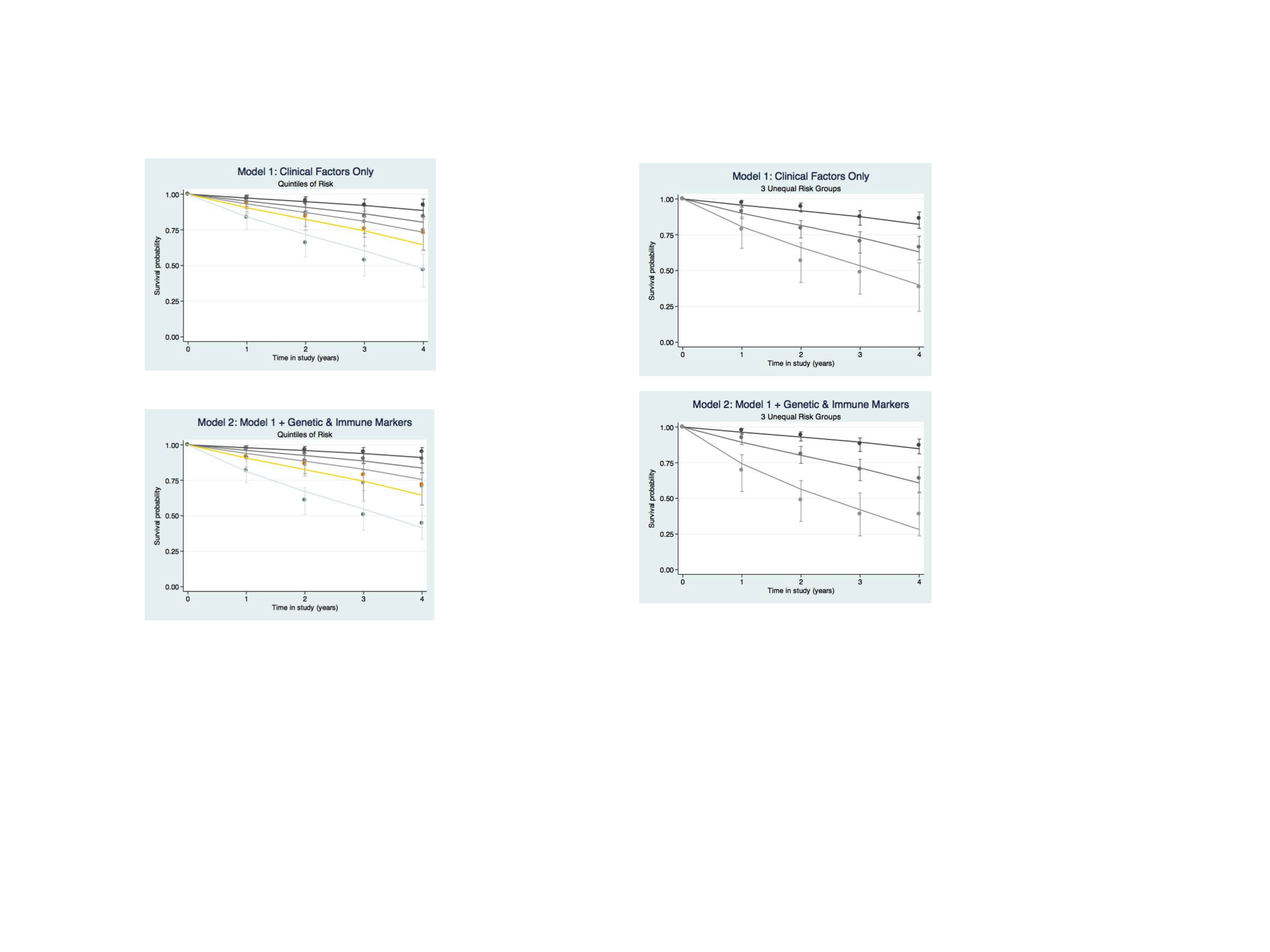

Supplement: S2 Fig — a)Left panel: Equal-sized Quintiles of 3-year Risk in Model 1 (top) vs. Model 2 (bottom) Smooth lines represent predicted survival probabilities, and vertical capped lines denote Kaplan–Meier estimates with 95% confidence intervals. Five prognosis groups are plotted: the “Good” group (darkest lines) and the “Poor” group (palest lines) at the highest and lowest risk categories, respectively, with the other 3 in between. b)Right panel: 3 Unequal Risk Groups (Cut at the 25th and the 75th Percentiles of the Failure Times) Smooth lines represent predicted survival probabilities, and vertical capped lines denote Kaplan–Meier estimates with 95% confidence intervals. Three prognosis groups are plotted: the “Good” group (darkest lines), the “Intermediate” group (medium-dark lines), and the “Poor” group (paler lines). Using unequal sized risk groups allows identification of individuals with the most extreme prognosis [41]. Model 1 included the following clinical predictors: sex, current alcohol use, HIV viral load, baseline APRI, HCV genotype 3 and ageModel 2 included Model 1 predictors and the following: genetic marker at IFNL rs8099917 and 5 immune markers IL-8, sICAM-1, RANTES, hsCRP, and sCD14. Abbreviations: IL-8, interleukin-8; sICAM-1, soluble intercellular adhesion molecule 1; RANTES, Regulated upon Activation, Normal T cell Expressed and Secreted protein; sCD14, soluble CD14; hsCRP high-sensitivity C-reactive protein. (TIF) [file pone.0176282.s006.tif]
